# Longitudinal Changes in Diagnostic Accuracy of a Differential Diagnosis List Developed by an AI-Based Symptom Checker: Retrospective Observational Study

**DOI:** 10.2196/53985

**Published:** 2024-05-17

**Authors:** Yukinori Harada, Tetsu Sakamoto, Shu Sugimoto, Taro Shimizu

**Affiliations:** 1 Department of Diagnostic and Generalist Medicine Dokkyo Medical University Shimotsuga Japan; 2 Department of General Medicine Nagano Chuo Hospital Nagano Japan; 3 Department of Medicine (Neurology and Rheumatology) Shinshu University School of Medicine Matsumoto Japan

**Keywords:** atypical presentations, diagnostic accuracy, diagnosis, diagnostics, symptom checker, uncommon diseases, symptom checkers, uncommon, rare, artificial intelligence

## Abstract

**Background:**

Artificial intelligence (AI) symptom checker models should be trained using real-world patient data to improve their diagnostic accuracy. Given that AI-based symptom checkers are currently used in clinical practice, their performance should improve over time. However, longitudinal evaluations of the diagnostic accuracy of these symptom checkers are limited.

**Objective:**

This study aimed to assess the longitudinal changes in the accuracy of differential diagnosis lists created by an AI-based symptom checker used in the real world.

**Methods:**

This was a single-center, retrospective, observational study. Patients who visited an outpatient clinic without an appointment between May 1, 2019, and April 30, 2022, and who were admitted to a community hospital in Japan within 30 days of their index visit were considered eligible. We only included patients who underwent an AI-based symptom checkup at the index visit, and the diagnosis was finally confirmed during follow-up. Final diagnoses were categorized as common or uncommon, and all cases were categorized as typical or atypical. The primary outcome measure was the accuracy of the differential diagnosis list created by the AI-based symptom checker, defined as the final diagnosis in a list of 10 differential diagnoses created by the symptom checker. To assess the change in the symptom checker’s diagnostic accuracy over 3 years, we used a chi-square test to compare the primary outcome over 3 periods: from May 1, 2019, to April 30, 2020 (first year); from May 1, 2020, to April 30, 2021 (second year); and from May 1, 2021, to April 30, 2022 (third year).

**Results:**

A total of 381 patients were included. Common diseases comprised 257 (67.5%) cases, and typical presentations were observed in 298 (78.2%) cases. Overall, the accuracy of the differential diagnosis list created by the AI-based symptom checker was 172 (45.1%), which did not differ across the 3 years (first year: 97/219, 44.3%; second year: 32/72, 44.4%; and third year: 43/90, 47.7%; *P*=.85). The accuracy of the differential diagnosis list created by the symptom checker was low in those with uncommon diseases (30/124, 24.2%) and atypical presentations (12/83, 14.5%). In the multivariate logistic regression model, common disease (*P*<.001; odds ratio 4.13, 95% CI 2.50-6.98) and typical presentation (*P*<.001; odds ratio 6.92, 95% CI 3.62-14.2) were significantly associated with the accuracy of the differential diagnosis list created by the symptom checker.

**Conclusions:**

A 3-year longitudinal survey of the diagnostic accuracy of differential diagnosis lists developed by an AI-based symptom checker, which has been implemented in real-world clinical practice settings, showed no improvement over time. Uncommon diseases and atypical presentations were independently associated with a lower diagnostic accuracy. In the future, symptom checkers should be trained to recognize uncommon conditions.

## Introduction

Diagnostic errors are a significant global patient safety issue [1]. In outpatient settings, diagnostic errors are evident in 1%-5% of cases [2-5]. Notably, the risk of such errors increases for outpatients unexpectedly admitted shortly after their initial visit [6,7]. The most common factors contributing to diagnostic errors in outpatient settings include problems with data integration, interpretation, and differential diagnosis [3,5,8-10]. To address this, the integration of diagnostic decision support systems, such as differential diagnosis generators, into clinical practice is recommended [11].

Differential diagnosis generators produce possible differential diagnoses by processing clinical information through algorithms, thereby supporting clinicians by reducing the likelihood of overlooking possible diagnoses and countering the cognitive biases inherent to the diagnostic process [12]. Early deployment of differential diagnosis generators can augment an existing list of differential diagnoses, increasing the odds of including the correct diagnosis [13], and can prompt more thorough history taking [14]. Therefore, current symptom checkers—generators that produce differential diagnoses based on the inputs from patients themselves before they encounter a clinician—are potentially promising tools to reduce diagnostic errors. Indeed, some symptom checkers have already been used in clinical practice [15-17] and even in national health services, such as the National Health Service 111 system in the United Kingdom [18].

Given that modern artificial intelligence (AI) is designed to be dynamic and to evolve according to real-world data [19], one might expect the performance of AI-based symptom checkers to improve over time. Importantly, at the same time, a decline in the performance of AI by feedback with data from different populations and settings is also possible. Monitoring such a shift and drift of AI performance is required to use AI-based symptom checkers effectively and safely [19,20]. However, their developers do not usually disclose the data, such as how AI algorithms changed and what types of clinical indicators improved. One set of studies using the same sets of clinical vignettes found that the diagnostic accuracy of symptom checkers improved from 2015 to 2020 [21]. However, because these case vignettes are publicly available, the developers may have trained symptom checker algorithms using these cases. Therefore, it remains unknown whether symptom checkers improve their diagnostic performance over time [12]. Moreover, because clinical vignettes have been found to have considerable inherent limitations when used to assess diagnostic accuracy in comparison with real-world data [22], longitudinal evaluations of the performance of symptom checkers in the real world are needed.

Concerns have arisen regarding the low diagnostic accuracy of current symptom checker output, which often lags behind that of physicians [12,17,23]. Inaccurate initial diagnoses can be detrimental, steering clinicians toward errors [24]. One major hurdle in the accuracy of symptom checker outputs is patient input variability. Differences in symptom interpretation, clinical literacy, input sequencing, and symptom listings can profoundly influence the quality of a symptom checker’s output [12,25]. Another challenge is the disparity between simulated and real-world data. Previous research has indicated a diminished accuracy of symptom checker output when applied to real cases instead of fictional vignettes [23]. This could be attributed to the fact that crafted vignettes often provide typical presentations [25], whereas real cases include more atypical presentations and may contribute to diagnostic errors [26]. Therefore, symptom checkers should be trained using real-world patient data, covering a diverse range of cases and including atypical presentations, to improve their accuracy [12,25]. The real-world application and refinement of these tools after development are crucial.

Therefore, this study was conducted to assess the changes in the accuracy of differential diagnosis lists created by AI-based symptom checkers in the real world. This paper defined AI-based symptom checkers as those using contemporary machine learning models.

The contributions of our proposed work are summarized as follows: (1) we provide the data of 3-year longitudinal changes in the diagnostic performance of contemporary machine learning–based symptom checkers and (2) we also provide factors related to the diagnostic performance of AI-based symptom checkers.

## Methods

### Study Design and Participants

**Figure 1:**
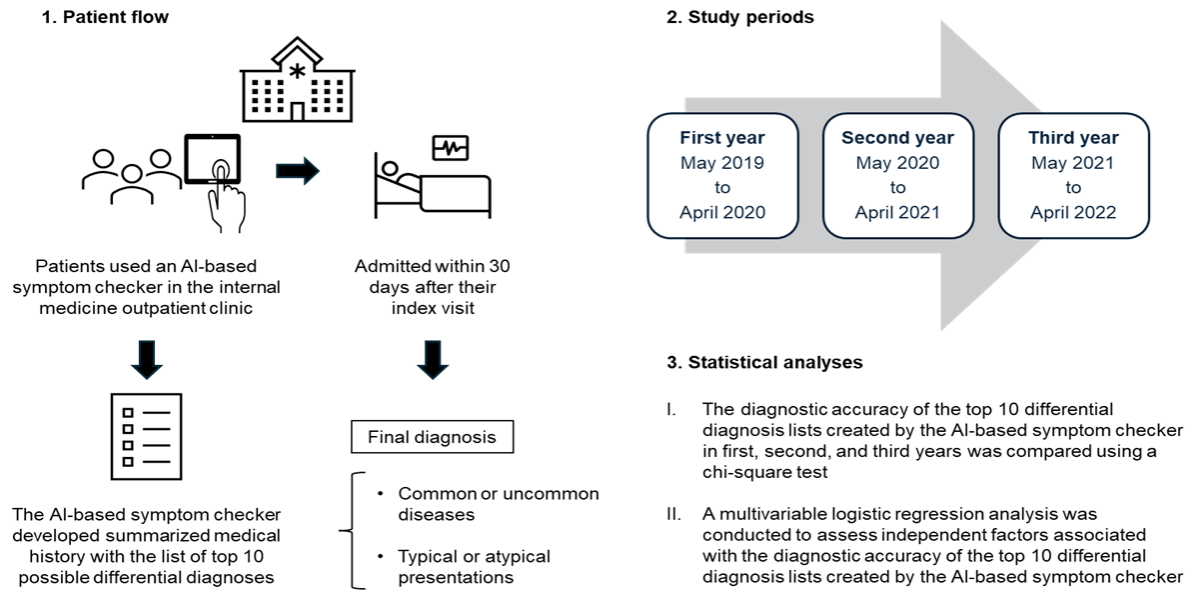
Overview of the study. This study included patients who visited the internal medicine outpatient clinic at a community hospital without an appointment between May 2019 and April 2022 and were admitted within 30 days after their index visit. This study included only patients who used an AI-based symptom checker that identified 10 possible differential diagnoses at the index visit. The final diagnoses were categorized into common or uncommon diseases, and clinical presentations were categorized into typical or atypical. The change in the diagnostic accuracy of the AI-based symptom checker over 3 years was assessed by using a chi-square test by dividing the study duration into 3 periods: from May 2019 to April 2020 (first year), from May 2020 to April 2021 (second year), and from May 2021 to April 2022 (third year). A multivariable logistic regression analysis was conducted to assess independent factors with a diagnostic accuracy of the top 10 differential diagnosis lists created by the AI-based symptom checker. AI: artificial intelligence.

This was a single-center, retrospective, observational study. Patients who visited the internal medicine outpatient clinic at Nagano Chuo Hospital without an appointment between May 1, 2019, and April 30, 2022, and who were then admitted within 30 days after their index visit were considered eligible. We set the inclusion criteria because admission within 30 days after the index visit was considered a useful option to capture the patients with a high risk of diagnostic errors [27-31]; diagnostic decision support systems are particularly needed for these population. We included only patients who used an AI-based symptom checker that identified 10 possible differential diagnoses (Ubie Inc) at the index visit and excluded patients for whom the AI-based symptom checker produced less than 10 differential diagnoses, whose diagnosis was not confirmed, and who were admitted for a reason unassociated with their index visit complaint. For patients who used the AI-based symptom checker multiple times at different outpatient visits or who were admitted twice or more, we included only data from the first outpatient visit and admission (others were excluded as duplicates). An overview of this study is shown in Figure 1.

### Ethical Considerations

The study complied with the principles of the Declaration of Helsinki. The research ethics committee of Nagano Chuo Hospital approved this study (NCR202208) and waived the requirement for written informed consent from the participants because of the opt-out method used in this study. We informed the participants by providing detailed information about the study in the outpatient waiting area at Nagano Chuo Hospital and on the hospital’s website. The study data are deidentified. There was no compensation for the participants.

### AI-Based Symptom Checker

Details of the AI-based symptom checkers assessed in this study have been described previously [7,32]. In brief, the AI-based symptom checker converted the data entered by patients on tablet terminals into medical terms. Patients entered their background information, such as age, sex, and chief complaint, as a free text on a tablet in the waiting room. This AI-based symptom checker asked approximately 20 questions, one by one, tailored to the patient. Based on the previous answers of the same patient, the questions were optimized to generate the most relevant list of potential differential diagnoses. The hospital staff at Nagano Chuo Hospital provided support to the patients when they found it difficult to input information independently. Physicians could view the entered data as a summarized medical history with the top 10 possible differential diagnoses along with their ranks. According to the developer’s website, this AI-based symptom checker improved quality through feedback from more than 1500 medical institutions. However, we could not show the mathematical expression and algorithm of the machine learning model because the developer did not disclose a detailed machine learning methodology.

### Data Collection

We retrospectively collected data from the patients’ electronic health records. The following data were collected: date of the index visit, age, sex, medical history recorded by the AI-based symptom checker (including chief complaints, history of present illness, past medical history, family history, and social history), 10 differential diagnoses developed by the AI-based symptom checker, and the final diagnosis. The final diagnosis was judged independently by 2 researchers (YH and SS) based on the descriptions in the medical records, and disagreements were resolved through discussion. Final diagnoses were coded by the first author (YH) using the *ICD-11* (*International Classification of Diseases, 11th Revision*) codes. Final diagnoses were further categorized into common or uncommon diagnoses based on whether the incidence was more than 1 in 2000 (common disease) or not (uncommon disease) [33]; unclear cases were judged by 2 researchers (YH and T Sakamoto) through discussion. According to the final diagnosis and medical history created by the AI-based symptom checker, 2 researchers (YH and T Sakamoto) independently judged all cases as typical or atypical, and conflicts were resolved by discussion.

### Primary Outcome

The primary outcome measure was the accuracy of the differential diagnosis list created using the AI-based symptom checker. The accuracy of the differential diagnosis list created by the AI-based symptom checker was defined as the presence of the final diagnosis in the list of 10 differential diagnoses created by the AI-based symptom checker. Two researchers (YH and T Sakamoto) independently judged the accuracy of the differential diagnosis list created by the AI-based symptom checker, and conflicts were resolved through discussion. The accuracy of the AI over 3 years was also assessed in the following subgroups: age 65 years and older and younger than 65 years, men and women, single and multiple chief complaints, common and uncommon disease, and typical and atypical presentation.

### Statistical Analysis

Continuous or ordinal data are presented as mean and SD or median and quantiles and compared using a 2-tailed *t* test, *U* test, or ANOVA. Categorical or binary data are presented as numbers and percentages and compared using the chi-square or Fisher exact test. To assess the change in the diagnostic accuracy of the AI-based symptom checker over 3 years, we compared the accuracy of the differential diagnosis lists created by the AI-based symptom checker using a chi-square test by dividing the study duration into 3 periods: from May 1, 2019, to April 30, 2020 (first year); from May 1, 2020, to April 30, 2021 (second year); and from May 1, 2021, to April 30, 2022 (third year). We calculated 108 patients as the minimum required sample size based on an **α** error of .05, power of 0.80, effect size of 0.30 (medium), and degrees of freedom of 2. We also created a multivariable logistic regression model that included the correctness of the differential diagnosis list created by the AI-based symptom checker as an independent variable and the visit year (first, second, and third year), age (as a continuous variable), sex (male or female), typicality of presentation (typical or atypical), and commonality of final diagnosis (common or uncommon) as dependent variables; these variables were selected as confounders because they were considered to be associated with the accuracy of the differential diagnosis list created by the AI-based symptom checker. *P* values below .05 were considered significant. All statistical analyses were performed using R (version 4.1.0; R Foundation for Statistical Computing).

## Results

### Baseline Characteristics

Of the 484 eligible cases, 103 were excluded (duplication: 20, admission unrelated to the index visit: 9, no final diagnosis: 18, and AI produced less than 10 differential diagnoses: 56). Therefore, 381 cases were finally included in the analysis. The mean age was 68 (SD 18) years, and 205 (53.8%) were men. In total, 174 (45.7%) patients inputted more than 1 complaint. Diseases of the digestive system were the most common final diagnosis category (n=128, 33.6%), followed by diseases of the circulatory system (n=55, 14.4%), respiratory system (n=44, 11.5%), neoplasms (n=42, 11%), and infectious or parasitic diseases (n=26, 6.8%). Regarding commonality and typicality, 257 (67.5%) were common diseases, and 298 (78.2%) were typical presentations. Typical presentation of common disease was the most common group (n=205, 53.8%), followed by typical presentation of uncommon disease (n=93, 24.4%), atypical presentation of common disease (n=52, 13.6%), and atypical presentation of uncommon disease (n=31, 8.1%). The number of patients was higher in the first year than in the second and third years (Table 1) due to the COVID-19 pandemic. Although there was a significant difference in age, no significant differences were observed in other baseline characteristics among the 3 groups.

**Table 1:** Baseline characteristics of patients who visited the internal medicine outpatient clinic at Nagano Chuo Hospital without an appointment and then admitted within 30 days for 3 years from May 2019 to April 2022.

	First year^a^ (n=219)	Second year^b^ (n=72)	Third year^c^ (n=90)	*P* value
Age (years), mean (SD)	70 (18)	63 (15)	64 (17)	.002
Men, n (%)	114 (52.1)	40 (55.6)	51 (56.7)	.72
Multiple chief complaints, n (%)	104 (47.5)	32 (44.4)	38 (42.2)	.68
Common disease, n (%)	146 (66.7)	45 (62.5)	66 (73.3)	.32
Typical presentation, n (%)	164 (74.9)	60 (83.3)	74 (82.2)	.18

^a^The first year was from May 1, 2019, to April 30, 2020.

^b^The second year was from May 1, 2020, to April 30, 2021.

^c^The third year was from May 1, 2021, to April 30, 2022.

### Primary Outcome

Overall, the final diagnosis was observed in the top 10 differential diagnosis lists created by the AI-based symptom checker in 172 (45.1%) patients. The accuracy of the differential diagnosis list created by the AI-based symptom checker did not significantly differ among the 3 years (first year: 97/219, 44.3%; second year: 32/72, 44.4%; and third year: 43/90, 47.7%; *P*=.85). There was also no significant difference in the accuracy of AI differential diagnosis among the 3 years in the subgroups (Table 2). In the subgroups with uncommon diseases and atypical presentations, the correct rate of the AI differential diagnosis list was <30%. Some examples of cases with uncommon diseases and atypical presentations are shown in Multimedia Appendix 1.

**Table 2:** Proportion of patients with a correct diagnosis included in the top 10 differential diagnosis list generated by artificial intelligence in patients who visited the internal medicine outpatient clinic at Nagano Chuo Hospital without an appointment and then admitted within 30 days for 3 years from May 2019 to April 2022.

	Total (N=381), n/N (%)	First year^a^ (n=219), n/N (%)	Second year^b^ (n=72), n/N (%)	Third year^c^ (n=90), n/N (%)	*P* value
Overall accuracy	172/381 (45.1)	97/219 (44.3)	32/72 (44.4)	43/90 (47.7)	.85
Age ≥65 years	110/243 (45.3)	69/159 (43.4)	15/35 (42.9)	26/49 (53.1)	.47
Age <65 years	62/138 (44.9)	28/60 (46.7)	17/37 (45.9)	17/41 (41.5)	.87
Men	102/205 (49.8)	53/114 (46.5)	20/40 (50)	29/51 (56.9)	.47
Women	70/176 (39.8)	44/105 (41.9)	12/32 (37.5)	14/39 (35.9)	.77
Single chief complaint	103/207 (49.8)	53/115 (46.1)	23/40 (57.5)	27/52 (51.9)	.43
Multiple chief complaints	69/174 (39.7)	44/104 (42.3)	9/32 (28.1)	16/38 (42.1)	.34
Common disease	142/257 (55.3)	79/146 (54.1)	27/45 (60)	36/66 (54.5)	.78
Uncommon disease	30/124 (24.2)	18/73 (24.7)	5/27 (18.5)	7/24 (29.2)	.67
Typical presentation	160/298 (53.7)	88/164 (53.7)	29/60 (48.3)	43/74 (58.1)	.53
Atypical presentation	12/83 (14.5)	9/55 (16.4)	3/12 (25)	0/16 (0)	.10

^a^The first year was from May 1, 2019, to April 30, 2020.

^b^The second year was from May 1, 2020, to April 30, 2021.

^c^The third year was from May 1, 2021, to April 30, 2022.

### Logistic Regression Model

In the multivariate logistic regression model, the year of the index visit was not significantly associated with whether the final diagnosis was included in the top 10 differential diagnosis lists created by the AI-based symptom checker (Table 3). By contrast, in the multivariate logistic regression model, the commonality of disease and typicality of presentation were significantly associated with the accuracy of the differential diagnosis list created by the AI-based symptom checker.

**Table 3:** A logistic regression model for whether the correct diagnosis was included in the differential diagnosis list generated by artificial intelligence in patients who visited the internal medicine outpatient clinic at Nagano Chuo Hospital without an appointment and then admitted within 30 days.

Variables			OR^a^ (95% CI)		*P* value
**Year of visit**					
	Second year^b^ (reference: first year^c^)	0.84 (0.45-1.54)		.57	
	Third year^d^ (reference: first year)	0.88 (0.51-1.54)		.67	
Age (for 1-year increase)			0.99 (0.98-1.00)		.16
Men (reference: women)			1.42 (0.92-2.30)		.11
Multiple complaints (reference: single complaint)			0.70 (0.44-1.11)		.13
Common disease (reference: uncommon disease)			4.13 (2.50-6.98)		<.001
Typical presentation (reference: atypical presentation)			6.92 (3.62-14.2)		<.001

^a^OR: odds ratio.

^b^The second year was from May 1, 2020, to April 30, 2021.

^c^The first year was from May 1, 2019, to April 30, 2020.

^d^The third year was from May 1, 2021, to April 30, 2022.

## Discussion

### Principal Results

In this study, at a community hospital in Japan, a 3-year longitudinal assessment of the performance of an AI-based symptom checker showed no change in the diagnostic accuracy of its differential diagnosis lists in outpatients admitted within 30 days of their index visit. In the exploratory subgroup and multivariate logistic regression analyses, the commonality of disease and typicality of presentation were significantly associated with the accuracy of the differential diagnosis list created by the AI-based symptom checker.

### Implications of the Study

This study suggests that current AI-based symptom checkers used in the real world may not improve their diagnostic performance over time. In this study, no improvement in the diagnostic accuracy of AI was observed, even in the common disease and typical presentation subgroups. Machine learning, using data with reliable teaching labels, is required to improve the accuracy of AI-based symptom checkers. However, patients may not always be able to accurately provide their final diagnosis, which may prevent effective machine learning. In addition, even if symptom checkers are used in health care facilities, reliable feedback may not be guaranteed because of diagnostic uncertainty, low diagnostic quality, and care fragmentation. The results of this study indicate that the developers and users of AI-based symptom checkers should be more responsible for improving the diagnostic quality of AI-based symptom checkers by providing reliable feedback on diagnostic labels.

There can be another perspective for this study’s results. We assumed that the performance of the AI-based symptom checker did not improve over time based on the result that the diagnostic accuracy did not change. However, it is possible that the developer also set indicators other than diagnostic accuracy, such as the impact of service use, clinical and cost-effectiveness, and patient satisfaction, to improve the algorithm of the AI-based symptom checker [17]. Balancing the different outcomes may limit the increase in diagnostic accuracy. In addition, since we do not know the ideal and theoretical upper limit of diagnostic accuracy in specific clinical contexts with some restrictions, it is also possible that some AI-based symptom checkers’ diagnostic accuracy has already reached the theoretical upper limit of their performance. For example, minimizing questions to save time may reduce the diagnostic performance. Indeed, our previous study showed that physicians’ diagnostic accuracy was only 56% when reading the information taken by the same AI-based symptom checker used in this study [34]. Therefore, the judgment that no improvement in diagnostic accuracy was observed in this study may be unfair. We need a standard method with clear indicators for an unbiased and fair evaluation of the improvement of the performance of AI-based symptom checkers.

### Comparison With Prior Work

Longitudinal comparisons of the diagnostic performance of symptom checkers in the real world are scarce; however, several studies have assessed changes in the diagnostic accuracy of symptom checkers using clinical vignettes. According to Schmieding et al [21], the rate of correct diagnoses listed among the top 10 differential diagnoses of symptom checkers was at least 15% higher in 2020 than in 2015 using the same clinical vignettes. In contrast, other studies suggested that the diagnostic accuracy of symptom checkers did not change from 2015 to 2020 when using some of the new vignettes [21,35]. Considering these and our study results, the diagnostic accuracy of symptom checkers may be improved for prototypical or standardized patients; however, because there are many variants of demographic patterns and clinical presentations in the real world, slight improvements may not result in the overall improvement of diagnostic accuracy.

In this study, the diagnostic accuracy of the AI-based symptom checker for uncommon diseases was approximately 30% lower than that for common diseases; similarly, approximately 40% lower diagnostic accuracy was observed for atypical presentations than for typical presentations. The diagnostic accuracy of symptom checkers may depend on the urgency of the clinical condition as well as common and uncommon conditions [17]. Indeed, a previous study also showed that the correct diagnosis was less frequently listed in the top 10 differential diagnoses of symptom checkers for uncommon diseases than for common diseases with a 60% difference (8% vs 68%) [35]. Our study provides evidence that atypical presentations, another aspect of uncommon conditions, may also negatively affect the diagnostic accuracy of symptom checkers. Uncommon diseases and atypical presentations are associated with a high risk of diagnostic error [26,36]. Through this perspective, our data indicate that current and future symptom checkers should be further trained with data on uncommon conditions, such as uncommon diseases and atypical presentations, to improve diagnostic quality in clinical practice. According to a previous study, symptom checkers can collect only 30% of all pertinent findings and are not good at collecting pertinent negative findings [37]. Considering that collecting pertinent findings is vital for diagnosing uncommon conditions, training with data on uncommon conditions and a system of high-quality feedback and reinforcement by expert diagnosticians are warranted for future symptom checkers.

Recent emerging generative AI-related tools such as ChatGPT (OpenAI Inc), a chatbot that uses a large language model, have been studied for their potential as new differential diagnosis generators. Several studies have demonstrated the high diagnostic accuracy of ChatGPT for simple to complex clinical cases using clinical vignettes and published case reports [38-40]. However, these studies input clinical information, including test results. Regarding symptom checking, while one study showed ChatGPT exhibited high accuracy in symptom checking for a broad range of diseases using the Mayo Clinic symptom checker as a benchmark [41], another study showed no difference in diagnostic accuracy between current symptom checkers and ChatGPT for patients with urgent or emergent clinical problems [15]. In addition, regarding ChatGPT, there is a concern that the near-infinite range of possible inputs and outputs prevents standardized regulations [15,42]. Furthermore, generative AI did not seem to overcome the problem of current symptom checkers that worsened diagnostic accuracy in cases of uncommon conditions [43]. Therefore, generative AI-related tools cannot be effective symptom checkers right now. However, compared to current symptom checkers, the diagnostic performance of generative AI-related tools can rapidly improve over time. Indeed, some studies showed that ChatGPT-4 outperformed ChatGPT-3.5 in diagnostic performance [38,43,44]. Therefore, generative AI-related tools may be a choice for diagnosis generators before patient-clinician encounters in the near future.

### Limitations

This study has some limitations. First, the modification details of the symptom checker model used in this study, including the type of machine learning methods used or manual updates used and the frequency at which the model was modified, remained unclear. Second, 3 years may not be appropriate for assessing contemporary machine learning model improvement since there is no standard time frame to assess the improvement of the machine learning model. However, considering that AI-related tools such as ChatGPT show rapid performance improvement, 3 years can be considered enough. Third, the COVID-19 pandemic may have affected our results due to low participants in the second and third years. Fourth, because this was a single-center retrospective study and we only included patients admitted within 30 days of the index outpatient visit, the results should be interpreted with caution regarding generalizability. Fifth, because there was no validated tool to assess the typicality of the presentation, which was assessed based on the information produced by the AI, the classification of typicality in this study may have been biased. This was also true for disease commonality, which could change if other criteria for uncommon diseases were applied.

### Conclusions

A 3-year single-center, retrospective, observational study of the diagnostic accuracy of differential diagnosis lists developed by an AI-based symptom checker, currently implemented in real-world clinical practice settings, showed no improvement over time. Uncommon diseases and atypical presentations were independently associated with lower diagnostic accuracy of the differential diagnosis lists generated by the AI-based symptom checker. In the future, symptom checkers should be trained to recognize uncommon conditions.

## Appendix

Multimedia Appendix 1 Examples of cases with uncommon diseases or atypical presentations.

## Abbreviations

AI: artificial intelligence
ICD-11: International Classification of Diseases, 11th Revision

## Data Availability

The data sets generated and analyzed during this study are available from the corresponding author on reasonable request.
