# Supplementary material for: Longitudinal Changes in Diagnostic Accuracy of a Differential Diagnosis List Developed by an AI-Based Symptom Checker: Retrospective Observational Study
Source: JMIR Form Res. 2024 May 17;8:e53985. doi: 10.2196/53985 (PMC11143391; doi:10.2196/53985)
Supplement: Multimedia Appendix 1 [file formative_v8i1e53985_app1.docx]

| Age and Sex | Chief complaints | Final diagnosis | Top 10 differential diagnosis lists created by the artificial intelligence-based symptom checker |
| --- | --- | --- | --- |
|  |  |  |  |
| **Atypical presentations** |  |  |  |
| 70’s man | Headache | Pneumonia | 1. Cluster headache  2. Infective endocarditis  3. Meningitis  4. Occipital neuralgia  5. Encephalitis/encephalopathy  6. Group A streptococcus infection  7. Hydrocephalus  8. Acute tonsillitis/pharyngitis  9. Giant cell arteritis  10. Sinusitis |
| 60’s man | Cough  Diarrhea  Loss of appetite | Acute myocardial infarction | 1. Asthma  2. Chronic obstructive pulmonary disease  3. Pulmonary fibrosis  4. Congestive heart failure  5. Pneumonia  6. Lung cancer  7. Sarcoidosis  8. Cough variant asthma  9. Atopic asthma  10. Tuberculosis |
| **Uncommon diseases** |  |  |  |
| 80’s man | Epigastric discomfort | Pneumatosis intestinalis | 1. Functional dyspepsia  2. Esophageal spasms  3. Stable angina  4. Aerophagia  5. Gastritis  6. Gastric cancer  7. Peptic ulcer disease  8. Sleep apnea syndrome  9. Reflux esophagitis  10. Systemic sclerosis |
| 60’s man | Jaundice | Ampullary carcinoma | 1. Anemia  2. Alcoholic hepatitis  3. Biliary tract cancer  4. Cirrhosis  5. Pancreatic cancer  6. Acute hepatitis  7. Gallstone  8. Acute cholecystitis  9. Primary biliary cholangitis  10. Liver tumor |
